# Au_147_(SPh)_30_(PPh_3_)_12_: A Geometrically Closed, but Electronically Open Triple‐Shell Icosahedral Gold Cluster and its Geometrically Open Counterpart

**DOI:** 10.1002/anie.202500586

**Published:** 2025-05-16

**Authors:** Markus Strienz, Andrei Poddelskii, Bridget K. Moll, Claudio Schrenk, Phillip S. Thomas, Andre Z. Clayborne, Andreas Schnepf

**Affiliations:** ^1^ Institut für Anorganische Chemie Universität Tübingen Auf der Morgenstelle 18 72076 Tübingen Germany; ^2^ Department of Chemistry and Biochemistry George Mason University 4400 University Drive MSN 3E2 Fairfax Virginia 22030 USA; ^3^ National Energy Research Scientific Computing Center (NERSC) Lawrence Berkeley National Laboratory 1 Cyclotron Rd Berkeley California 94720 USA

**Keywords:** Crystal structure, DFT‐calculations, EPR, Icosahedron, Metal cluster

## Abstract

The aesthetic platonic solids have been known since ancient times, and the structure of all five platonic solids is also found in chemical compounds. While gold sub‐nanometer clusters and gold nanoparticles with an icosahedral structure have been known for a long time to exist, a multi‐shell icosahedral gold cluster at the intermediate size between 13 and thousands of atoms has been elusive. Here we present the synthesis and crystallographic characterization of the first triple‐shell icosahedral metal cluster, Au_147_(SPh)_30_(PPh_3_)_12_ **1**. The gold core in **1** is stabilized by phosphines and thiolates, but surprisingly no staple motifs are formed. A second cluster, Au_146_(SPh)_30_(PPh_3_)_12_ **2**, cocrystallizes and is identified as having a closed electronic shell but can be considered as a geometrically open pendant of **1**. The unique clusters are characterized experimentally by EDX, UV/vis, DLS, and EPR and theoretically by quantum chemical calculations.

## Graphical abstract

The first triple‐shell icosahedral metal cluster, Au_147_(SPh)_30_(PPh_3_)_12_, is stabilized by phosphines and thiolates but does not show stable motifs. This cluster fills the gap between single‐shell clusters and nanoparticles in the form of an icosahedron. This geometrically closed but electronically open cluster co‐crystallizes with an Au_146_(SPh)_30_(PPh_3_)_12_ cluster, which is in contrast geometrically open but electronically closed. Both clusters provide a unique view of the transition between molecules and nanoparticles.

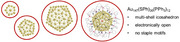

## Introduction

The transition between the molecular and bulk size regimes for gold clusters (and others) remains poorly understood. Using mass spectrometry on xenon atom clusters, Echt et al. demonstrated that the intensity of the masses corresponding to clusters containing 13, 19, 25, 55, 71, 87, and 147 xenon atoms is increased, and therefore these clusters are preferred over others of different sizes.^[^ ^1^ ^]^ However, there is no structural information available from mass spectrometry for these compounds.

In 1962, Mackay reported the assembly of icosahedral shells constructed from hard spheres.^[^ ^2^ ^]^ Thereby, the first shell is an icosahedron of 12 spheres arranged around a central sphere. Each of the 12 spheres is at a vertex of the icosahedron with its 20 triangles and 30 edges. A second shell comprising 42 spheres and a third shell comprising 92 spheres are constructed in such a way that the spheres are in contact along the fivefold axes. The number of spheres in each shell can be expressed by the equation 10*n* ^2^+2, with *n* representing the number of the shell. Consequently, single‐, double‐, and triple‐shell Mackay‐like icosahedra contain 13, 55, and 147 spheres, respectively, which is consistent with the numbers measured by Echt for the size of xenon clusters. Nevertheless, this does not constitute proof of their icosahedral structure, given that a cuboctahedral and an Ino‐decahedral arrangement of the atoms exhibits the same closed shell numbers and can be expressed by the same equation as for icosahedral structures. While a number of simulations suggest that the icosahedron is the most energetically favorable geometry for sizes up to a few thousand atoms, SCXRD measurements are the only clear evidence for structural determination.^[^ ^3^, ^4^, ^5^, ^6^ ^]^

**Figure 1:**
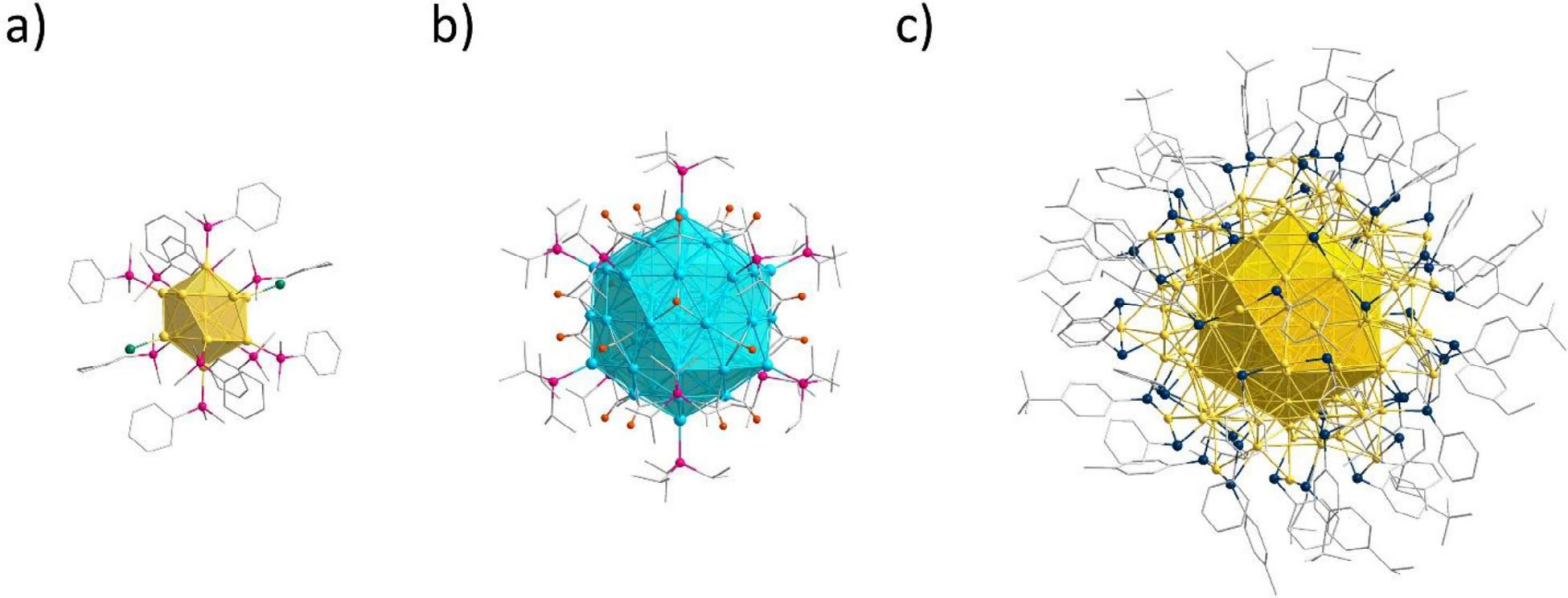
Structures of a) [Au_13_(PMe_2_Ph)_10_Cl_2_]^3+^, b) Pd_55_(P*^i^* Pr_3_)_12_(CO)_20_, and c) Au_133_(SPh*^t^* Bu)_52_. The icosahedral core is emphasized. Au (yellow), Pd (light blue), S (dark blue), Cl (green), P (pink), C (grey). Hydrogen atoms are omitted for clarity.

In 1981, Briant et al. synthesized and crystallized a compound containing 13 gold atoms.^[^ ^7^ ^]^ The 13 gold atoms are arranged in the form of an icosahedron, with a central gold atom surrounded icosahedrally by 12 gold atoms (Figure 1a). These 12 gold atoms are connected via direct gold–gold contacts. Therefore, this compound is a cluster, as defined by Cotton.^[^ ^8^ ^]^ To emphasize the topological metallic character of clusters, Schnöckel introduced the term “metalloid” to the nomenclature.^[^ ^9^ ^]^ A metalloid cluster is thereby a cluster with more metal–metal than metal‐ligand contacts, and there is a minimum of one naked gold atom, which is the central gold atom in this [Au_13_(PPh_2_Me)_10_Cl_2_]^3+^ cluster. Schmid et al. published the synthesis of a gold cluster consisting of 55 gold atoms in 1981.^[^ ^10^ ^]^ This cluster is in accordance with Echts measurements and could express the double‐shell icosahedron. While this cluster has not yet been crystallized, other characterization methods, such as Mössbauer spectroscopy, suggest a cuboctahedral rather than an icosahedral structure.^[^ ^10^ ^]^

This example underlines the importance of a single crystal for crystal structure analysis to clarify the shape, rather than relying solely on mass spectrometry experiments. The icosahedral derivative with 55 core atoms was synthesized for the first time by Dahl in 2016.^[^ ^11^ ^]^ The Pd_55_(P*^i^* Pr_3_)_12_(CO)_20_ cluster, with a double shell icosahedral Pd_55_ core, is coordinated by P*^i^* Pr_3_ ligands at each of the 12 vertices, while the 20 carbonyl groups bind each of the 20 faces (Figure 1b). The second double‐shell icosahedral cluster, Cu_43_Al_12_, is composed of a Cu_13_ core encircled by a bimetallic (Cu/Al)_42_ shell, where each aluminum atom binds a Cp* ligand.^[^ ^12^ ^]^ These two clusters, together with a recently published Fe_55_ cluster, are the only known multi‐shell clusters with an icosahedral shape.^[^ ^13^ ^]^ Nevertheless, a multitude of clusters have been synthesized that possess a single‐, double‐, or triple‐shell icosahedral core. However, all such clusters exhibit a disparate overall shape. Examples include the Au_133_(SPh*^t^* Bu)_52_ (Figure 1c) and Au_144_(SC_2_H_4_Ph)_60_ clusters.^[^ ^14^, ^15^, ^16^ ^]^ The development of high‐resolution electron microscopes has enabled the observation of the structure of nanoparticles that are larger in size than the aforementioned clusters. It is noteworthy that the icosahedron represents a common structural motif for these nanoparticles with sizes from 8 to 230 nm as well. These icosahedral nanoparticles are observed in a variety of materials, including transition metals such as gold, silver, platinum, and palladium, as well as alloyed nanoparticles comprising different elements.^[^ ^17^, ^18^ ^]^

The existence of icosahedral nanoparticles with thousands of atoms or more has thus been confirmed by high‐resolution electron microscopy. However, with the three exceptions of double‐shell icosahedra, there are no model compounds for multi‐shell icosahedral structures that bridge the gap between the aforementioned compounds and thus provide insight into the transition from molecular to metallic structure.

## Results and Discussion

**Figure 2:**
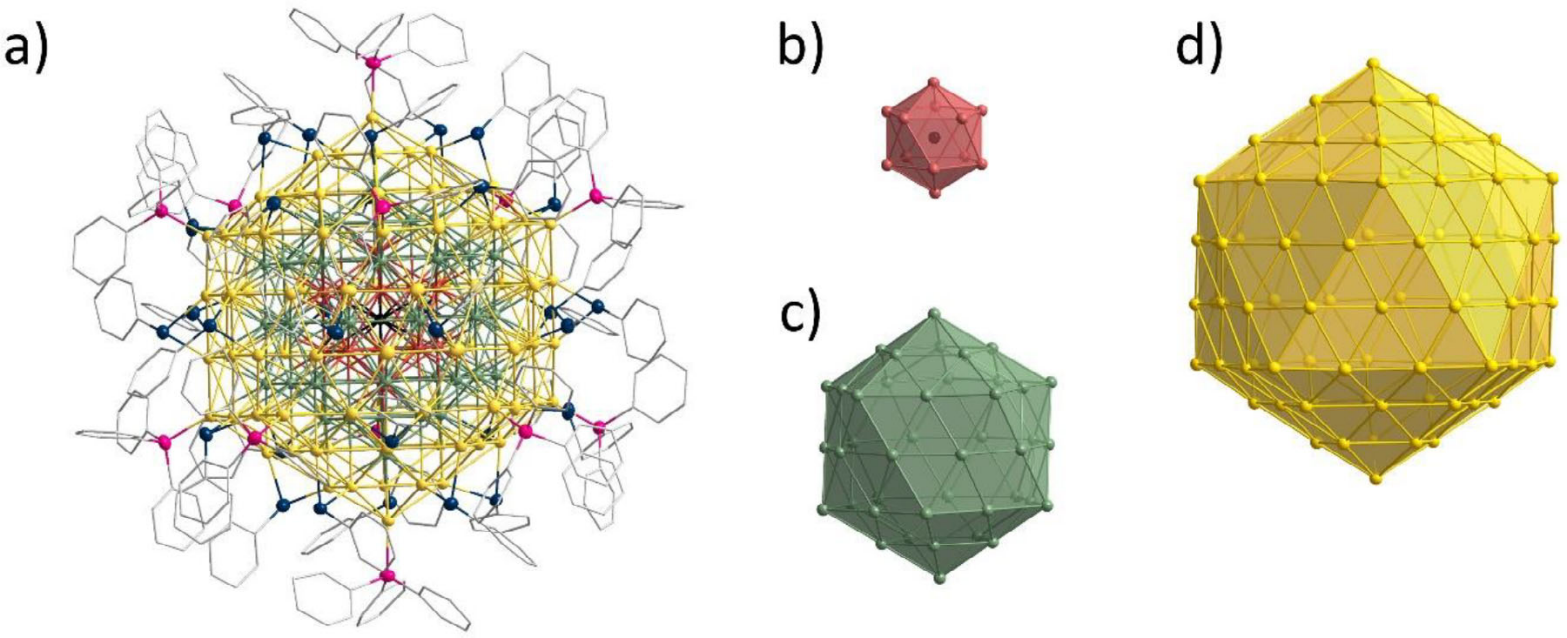
a) Molecular structure of **1** in the solid state. All atoms except for carbon are displayed as thermal ellipsoids with a 20% probability. Hydrogen atoms are omitted for clarity. **1** consists of three icosahedral shells, b) an Au_12_ shell (red) surrounding a central gold atom (black), c) a second Au_42_ shell (green), and d) a third Au_92_ shell (yellow). P (pink), S (blue), C (grey).

**Figure 3:**
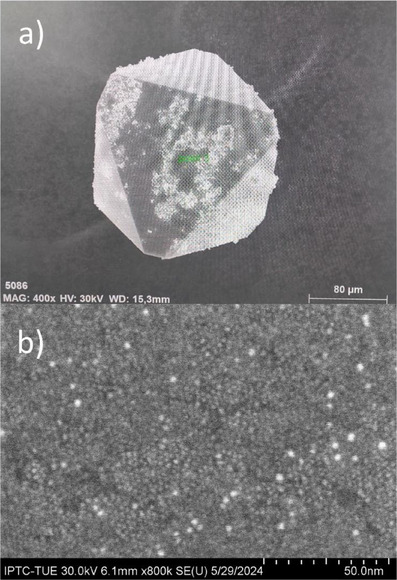
SEM image of a crystal at a) 400× magnification. The green mark is the EDX measurement point. b) 800 000× magnification. Individual clusters forming the single crystal can be seen as spheres.

The reduction of (Ph_3_P)AuSPh in THF (tetrahydrofuran) with L‐selectride (LiB*^s^* Bu_3_H) at room temperature gives the Au_147_(SPh)_30_(PPh_3_)_12_ cluster **1** (Figure 2a) (for a detailed synthesis, see Section S1.2). Crystallization of **1** leads to octahedral crystals in benzene in the trigonal crystal system with an R $\bar{3}$ space group after a few days at room temperature (Figure 3a). In addition to **1**, there is a second cluster, Au_146_(SPh)_30_(PPh_3_)_12_ **2** cocrystallizing. The structure of **2**, as well as the differences between it and **1**, will be discussed in detail after the initial presentation of **1**. The 147 gold atoms in **1** are arranged in a manner that a central gold atom is surrounded by three icosahedral shells (Figure 2b–d) consisting of 12, 42, and 92 gold atoms, respectively. Therefore, they fulfill Mackay's aforementioned definition of an icosahedral structure with geometrically closed shells.^[^ ^2^ ^]^ Consequently, **1** is the first triple‐shell Mackay‐like icosahedral cluster to be discovered.

The average distances between the gold atoms in an icosahedral shell increase with their size. The lowest lengths are found in the inner Au_12_ shell, with distances between the gold atoms of 289.8 ± 0.5 pm, increasing to 293.3 ± 1.9 pm in the Au_42_ shell and to 293.7 ± 6.5 pm in the third Au_92_ shell (Table 1). Furthermore, the gold–gold distances between the shells also increase from the center to the surface of the cluster. Thereby, gold–gold bond lengths of 275.7 ± 0.3 pm for the bonds between the central gold atom and the first shell, 280.5 ± 1.3 pm between the first and second shell, and 280.6 ± 3.3 pm between the second and the third shell are realized. Similar gold–gold distances and the increase in average bond distances with larger shells were observed in the icosahedral clusters Au_13_ and Au_133_ (Table 1).^[^ ^7^, ^16^ ^]^

**Table 1:** Comparison of the gold–gold distances in and between the different shells of **1** and the Au_13_, Au_133_, Au_144_, and (Au/Ag)_267_ clusters in pm.

	1	Au_13_	Au_133_	Au_144_	(Au/Ag)_267_
Center atom–shell 1	275.7 ± 0.3	276.9 ± 2.5	276.2 ± 0.8		268.4
Shell 1	289.8 ± 0.5	291.1 ± 2.9	289.6 ± 2.9	274.8 ± 0.3	282.1 ± 0,2
Shell 1–shell 2	280.5 ± 1.3		283 ± 3.4	286.5 ± 2.3	280.5 ± 2.1
Shell 2	293.3 ± 1.9		294.4 ± 7.1	291.4 ± 1.3	288.9 ± 2.7
Shell 2–shell 3	280.6 ± 3.3				286.4 ± 6.8
Shell 3	293.7 ± 6.5				291.5 ± 4.3

Shorter bond lengths are found in the cores of the Au_144_ and (Au/Ag)_267_ clusters.^[^ ^14^, ^19^ ^]^ This can be explained by the fact that the central atom position of the Au_144_ cluster is unoccupied, giving an Au_54_ core in contrast to the Au_55_ core of the Au_133_ cluster and **1**. The missing gold atom leads to a shrinking of the icosahedral Au_12_ shell. Therefore, the bond lengths within the icosahedral Au_12_ shell are only 274.8 ± 0.3 pm long and undercut the distances in **1** by 15 pm. The distortion of the gold atoms toward the center becomes smaller for the gold atoms in the larger shells, which is seen at similar distances for these shells in **1** and the Au_144_ cluster. The shorter distances in the core of the (Au/Ag)_267_ cluster, which has a bimetallic triple‐shell icosahedral core surrounded by 120 silver atoms, are most likely due to the two different elements in the core and the additional fourth shell.^[^ ^19^ ^]^

**Figure 4:**
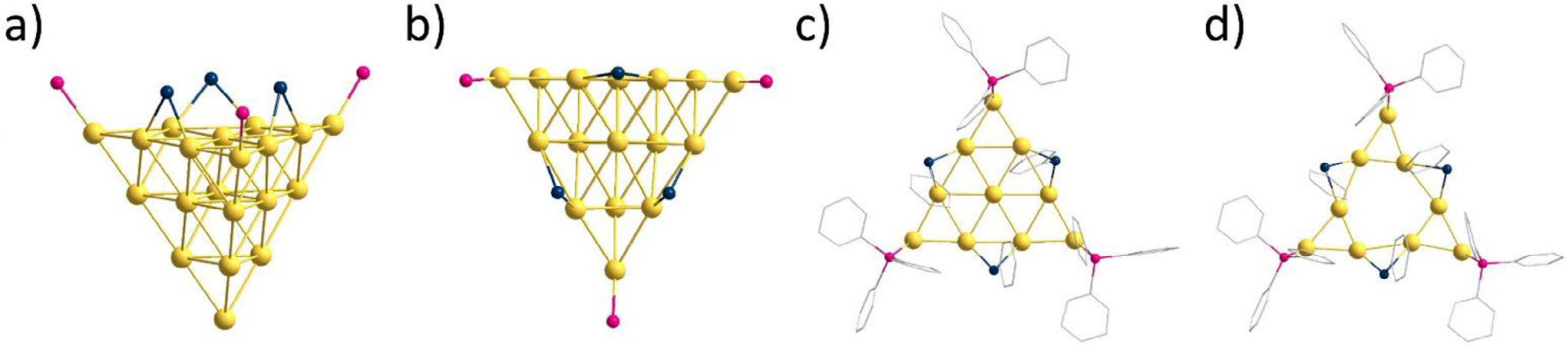
a) Side view of one of the 20 tetrahedra that make up **1**. b) Top view of one of the faces of **1**. c) One of the 20 triangles of the third shell of **1**. d) The central gold atom of the right triangle is missing, resulting in an Au_145_/Au_146_ cluster. Au (yellow), P (pink), S (blue), and C (grey). Hydrogen atoms are omitted for clarity.

The icosahedral arrangement of the 147 gold atoms in **1** can be subdivided into 20 tetrahedra, with one vertex of the tetrahedra situated at the center of the cluster. The other three vertices of the tetrahedra are the three gold atoms coordinated by a phosphine at the vertex of a triangular face of the icosahedron. Therefore, each of the 20 triangular surfaces of the icosahedron is also a face of one of the 20 tetrahedra. Consequently, each tetrahedron has four layers, containing 1, 3, 6, and 10 gold atoms, respectively (Figure 4a). Each layer corresponds to one of the three shells or the central atom, with a total of four layers in each tetrahedron. The four layers are arranged in such a way that the first and fourth layers are identical, resulting in an ABCA stacking sequence. This can be clearly seen in Figure 4b, in which the central gold atoms from layers a and d are congruent. The stacking in **1** is analogous to the stacking sequence observed in the cubic close‐packed (ccp) structure of elemental gold. The 20 tetrahedra are not ideal, as the angle between two vertices and the center of the icosahedron is 63.26°. Consequently, the packing density is lower than that of an ideal tetrahedron. The packing density for the icosahedral closed packing (icp) is dependent upon the number of shells and decreases from 72.59% for the first shell to 69.24% for the third shell when hard, identical‐sized spheres are utilized.^[^ ^2^ ^]^ Similar density values have been calculated for **1**, with a density of 72.44% for the first shell, a lower value of 68.1% for the second, and a slightly higher value of 69.89% for the third shell (for calculations, see Section S2.1).

The discrepancy between the observed and theoretical values for the third shell can be attributed to the binding of thiolates to these gold atoms. The radius of these oxidized gold atoms is smaller than that of the gold atoms in the core, which allows for a higher density. The packing density is less than that of the cubic closest packing, despite the identical stacking sequence within the tetrahedra. The distinction between the two structures lies in the octahedral vacancies that are present in the ccp lattice but absent in the icp lattice. The ABCABC stacking sequence is also found in the cuboctahedron, whose structure is actually a subset of the ccp lattice. Both polyhedra have the same number of spheres for their closed geometric structures and can be transformed into each other by splitting the squares of the cuboctahedron into two triangles to get an icosahedron, and vice versa. The cuboctahedron can grow further and become the fcc lattice. This is not possible for the icosahedron, whose growth along the 5‐fold axis results in a quasi‐crystal.^[^ ^20^ ^]^

X‐ray measurements give an occupation factor of 77.9% for two atom positions in the gold core (Section S2.2). This indicates that a second, metalloid gold cluster is co‐crystallizing, where a gold atom is missing at this position. Therefore, the probability of either an Au_146_ cluster co‐crystallizing is 44%, or an Au_145_ cluster co‐crystallizing is 22%. To better understand which core composition may exist, we look at the interplay of geometric shell closing and electronic shell closing. It is well‐known that typically compounds with an open electronic shell are less stable than those with a closed electronic shell. However, as in the case of **1**, the geometric shell can stabilize the system in lieu of a closed electronic shell. Thus, if one expects Au_146_(SPh)_30_(PPh_3_)_12_ **2** to only differ from **1** by removing one gold atom in the icosahedron, the closed electronic shell of **2** should be preferred over Au_145_. Further, there should be minimal change to the geometric shell closing of **2** with only one gold atom removed. Nevertheless, this conclusion is not definitive, and there is a possibility that the second compound is, in fact, Au_145_. The experimental control with mass spectrometry was unsuccessful due to the large mass of the clusters and their bad solubility (Section S9).

The aforementioned atom position with the lower occupation factor is in the middle of one of the triangles of the outer shell (Figure 4c,d). These gold atoms are the only gold atoms in the outer shell that are not bound by any ligand. Due to the missing gold atom, the bond lengths of **2** differ in the third shell since the missing gold atom causes a small shift of the surrounding gold atoms. All other positions are comparable, or the differences could not be resolved experimentally.

At this point it is worth mentioning that the double‐shell icosahedral core of the two clusters Au_133_ and Au_144_ also differs by one atom. However, this gold atom is located in the center of the core, with an occupied position for Au_133_ and an unoccupied position for Au_144_, and not on the surface as in **1** and **2**.

For both metalloid clusters **1** and **2**, each of the twelve vertices of the outer icosahedron is coordinated by a PPh_3_, where the phosphorus atom binds to the gold atom at the top of the vertex. The length of this bond is 227.5 pm and is consistent with other phosphine‐stabilized gold clusters.^[^ ^21^ ^]^ All 30 edges of the outer icosahedron are bridged by a phenylthiolate, with the sulfur binding to two gold atoms (Figure 4c). It is noteworthy that no staple motifs are observed in **1** and **2**, in contrast to the majority of thiolate stabilized clusters, where normally staple motifs of different sizes are observed. Thereby, larger clusters, such as the previously mentioned Au_144_(SC_2_H_4_Ph)_60_ and Au_133_(SPh*^t^* Bu)_52_, typically exhibit small S(R)‐Au‐S(R) units. On the other hand, smaller clusters such as Au_18_(SC_6_H_11_)_14_ feature longer Au_4_(SC_6_H_11_)_5_ staples.^[^ ^22^ ^]^ However, this structural motif is absent in **1** and **2**. Therefore, the icosahedral gold core is stabilized by 12 phosphines and 30 thiolates, where each thiolate bridges two gold atoms. The gold/phosphorus/sulfur ratio was also validated via EDX in a high‐precision manner (Section S3). The SEM pictures demonstrate the octahedral crystal shape (Figure 3a). At higher magnification, the individual clusters that make up the crystals become visible as small spheres (Figure 3b).

While the majority of gold clusters contain either thiolates or phosphines, which serve to stabilize the cluster, there are also promising attempts to stabilize the cluster with both. This can be achieved by the addition of thiolates to an existing phosphine‐stabilized cluster. The addition of thiolates to an Au_11_(PPh_3_)_7_Cl_3_ cluster results in the formation of a [Au_25_(PPh_3_)_10_(SR)_5_Cl_2_]^2+^ cluster, which is built from two Au_13_ icosahedra.^[^ ^23^ ^]^ The icosahedra are coordinated by phosphines and chlorides, while the linking of the two icosahedra is bridged by five thiolates. The reduction of (Ph_3_P)AuCl in the presence of a thiol results in the formation of an Au_108_S_24_(PPh_3_)_16_ cluster.^[^ ^24^ ^]^ The Au_108_ cluster is stabilized by 16 PPh_3_ ligands, with the sulfur forming unusual Au_4_S_4_ ring motifs. This indicates that phosphines may be a contributing factor leading to the synthesis of clusters with thiolates in a form other than staple motifs.

To observe the influence of the ligands and possibly increase the solubility of the clusters, we used gold precursors with slightly different phosphines ((*p*‐MePh)_3_P) and thiolates (*p*‐MePhS, *^t^* BuPhS, etc.). However, neither of these precursors produced a cluster similar to **1**, nor to another cluster. This emphasizes the importance of the right ligand system. The only change was observed when the synthesis was performed at lower temperatures. Upon reducing (Ph_3_P)AuSPh with L‐selectride at 0 °C, red crystals were observed in addition to the black crystals of **1** and **2** (Section S1.3).

**Figure 5:**
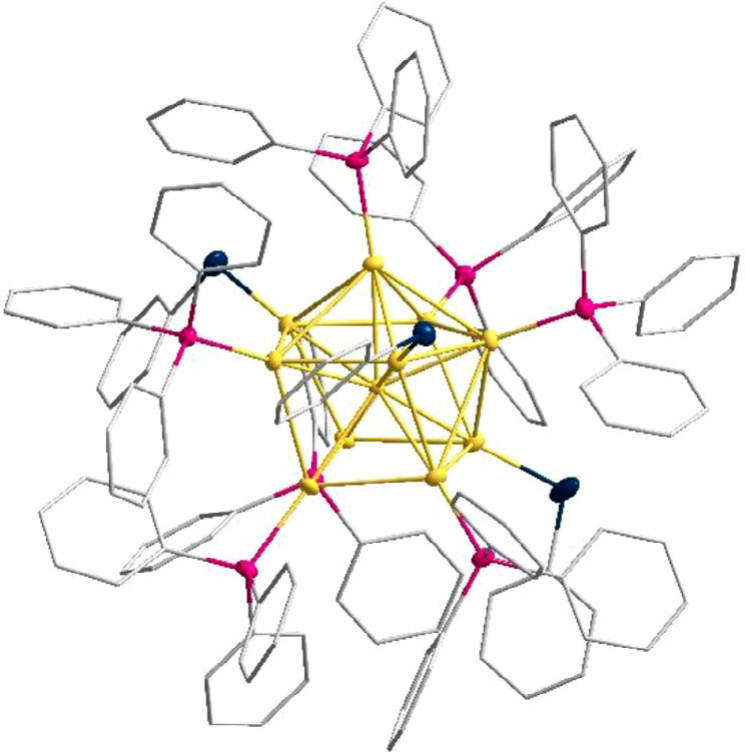
Molecular structure of **3** in the solid state. All atoms except for carbon are displayed as thermal ellipsoids with a 50% probability. Au (yellow), P (pink), S (black). Hydrogen atoms are omitted for clarity.

The red crystals consist of an Au_11_(PPh_3_)_7_(SPh)_3_ cluster **3**, crystallizing in the orthorhombic *P*2_1_2_1_2_1_ space group (Figure 5). The eleven gold atoms in **3** are arranged in the form of a centaur polyhedron, a combination of a cube and an icosahedron. All ten outer gold atoms are coordinated by a phosphine or a thiolate ligand, with only one naked gold atom in the center. This structural motif for gold atoms has been known for a long time and exists with different ligands (Au_11_L_7_X_3_; L = PPh_3_, PPh_2_Py,…; X = Cl, Br, I, SCN,…).^[^ ^21^, ^25^, ^26^, ^27^, ^28^ ^]^

However, the occurrence of **3** in this reaction system is remarkable, as thus all three clusters obtained from the same reaction exhibit the icosahedral motif. Consequently, **3** might be the seed for the formation of **1** and **2** in this reaction system, further enlightening the complex process of cluster and nanoparticle formation on an atomic scale. **1** is insoluble in many organic solvents, including pentane and toluene, and is weakly soluble in THF and 1,2‐difluorobenzene. However, **1** is soluble in DMI (1,3‐dimethylimidazolidin‐2‐one). The low solubility is most likely related to the phenyl ligands. The absorption spectrum of **1**, measured in DMI, shows two broad absorption bands at 390 and 532 nm (Section S7).

Thereby, the spectrum looks similar to that of the previously mentioned Au_133_ or other clusters in this size regime, with a main absorption at 500 nm. DLS measurements show a single signal at 9.1 nm (Section S5). While the cluster itself has a diameter of around 3 nm, as shown with SEM as well as from crystal structure analysis, 9 nm is plausible for the cluster with a coordinated solvent shell. Therefore, we can argue that the cluster is intact after solvation.

To gain insight into the stable nature of **1** and **2**, we carried out DFT calculations (Section S10). Often, the electronic structure of clusters with a gold core surrounded by ligands can be rationalized using electron counting or electronic shell models. For **1**, this results in a total of 117 electrons since it is known that phosphine ligands do not withdraw electrons.

**Figure 6:**
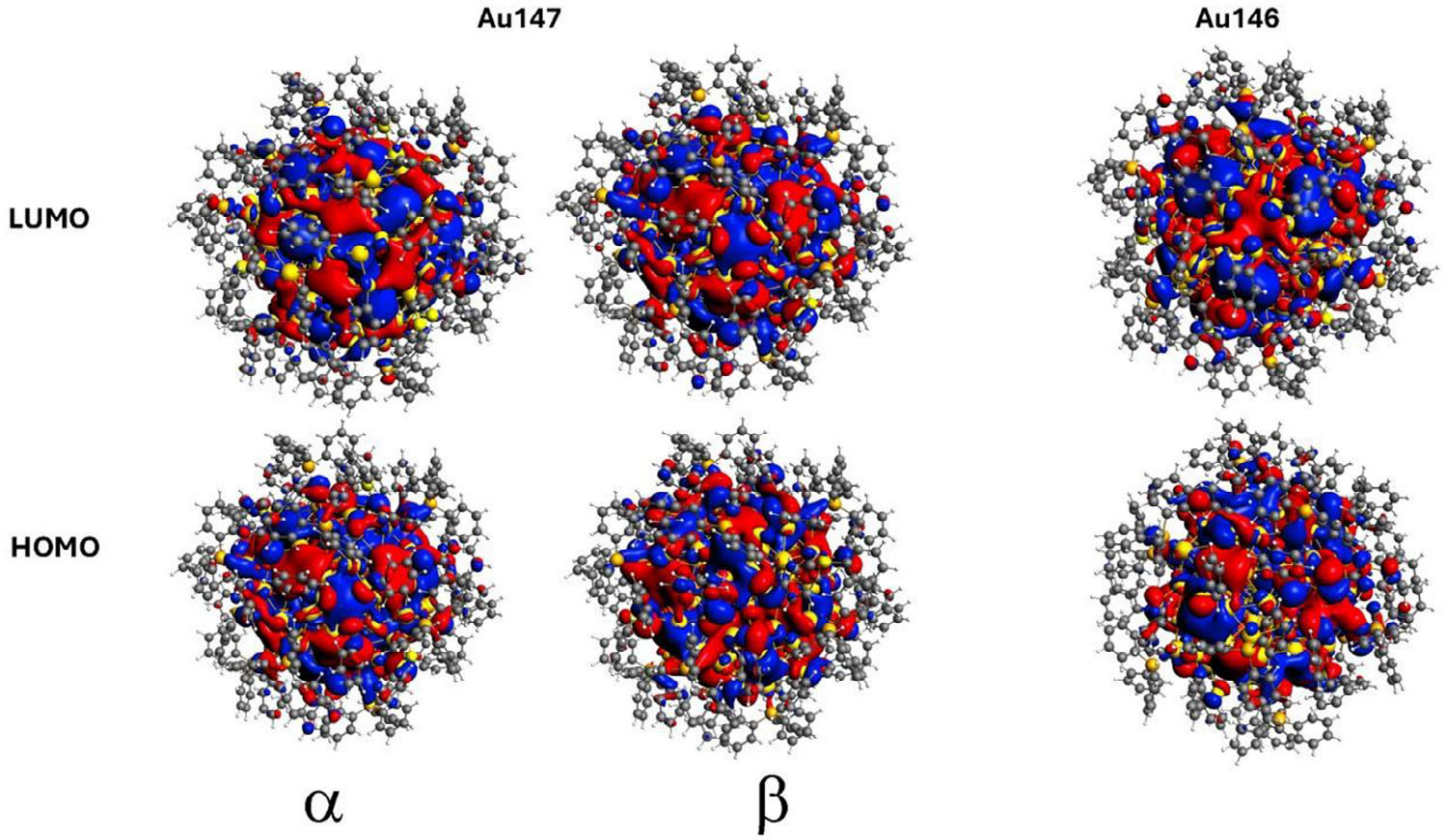
Image of the highest occupied orbital density for Au_146_ and Au_147_ clusters. The Au_147_ orbital density shows the spin‐up (α) and spin‐down (β) channels.

Given the spherical nature of **1**, one expects an open shell electron configuration: *1S^2^1P^6^1D^10^2S^2^1F^14^2P^6^1G^18^2D^10^3S^2^1H^22^2F^14^3P^6^1I^5^*. Interestingly, there is high symmetry observed in the orbitals of **1** and **2** (Figure 6), which exhibits *I*‐orbitals fitting to the assumed electron configuration.

It should be noted that though the I‐shell is not completely filled, there is one unpaired electron, resulting in a doublet for **1**. Often, an open shell configuration, as in **1,** results in a small HOMO‐LUMO gap (even with orbital splitting), with the possibility that each spin channel (e.g., spin‐up [α] or spin‐down [β]) may have a slightly larger gap than the HOMO–LUMO gap.

Here, we find the gap for Au_147_ to be 0.005 eV, while the α and β channels have gaps of 0.387 and 0.043 eV, respectively. This implies that by removing one gold atom, the Au_146_ system would have a closed shell and relatively larger gap. We find that the HOMO–LUMO gap for **2** (0.091 eV) is much larger than that of **1**. The small gap of **1** and larger gap found for **2** are expected; yet, these values may be an indicator that both clusters are approaching the bulk limit for nanoscale systems with both thiolate and phosphine ligands. It should be noted that the gaps for **1** and **2** are significantly smaller than those in Au_102_ (0.48 eV) and Au_144_ (1.67 eV), but in the same range as for Au_133_ (0.05 eV).^[^ ^29^ ^]^

Previous studies have shown that electronic shells of large gold systems such as Au_144_ can also be described through the symmetry lens. It is possible that **1**, having similar *I*‐orbitals to Au_144_, could have spherical symmetries around the Fermi level. Similarly, the combination of the icosahedral symmetry and packing of gold atoms in the core provides a geometric shell closing, enhancing the stability in the presence of an open (electronic) shell.^[^ ^30^ ^]^

**Figure 7:**
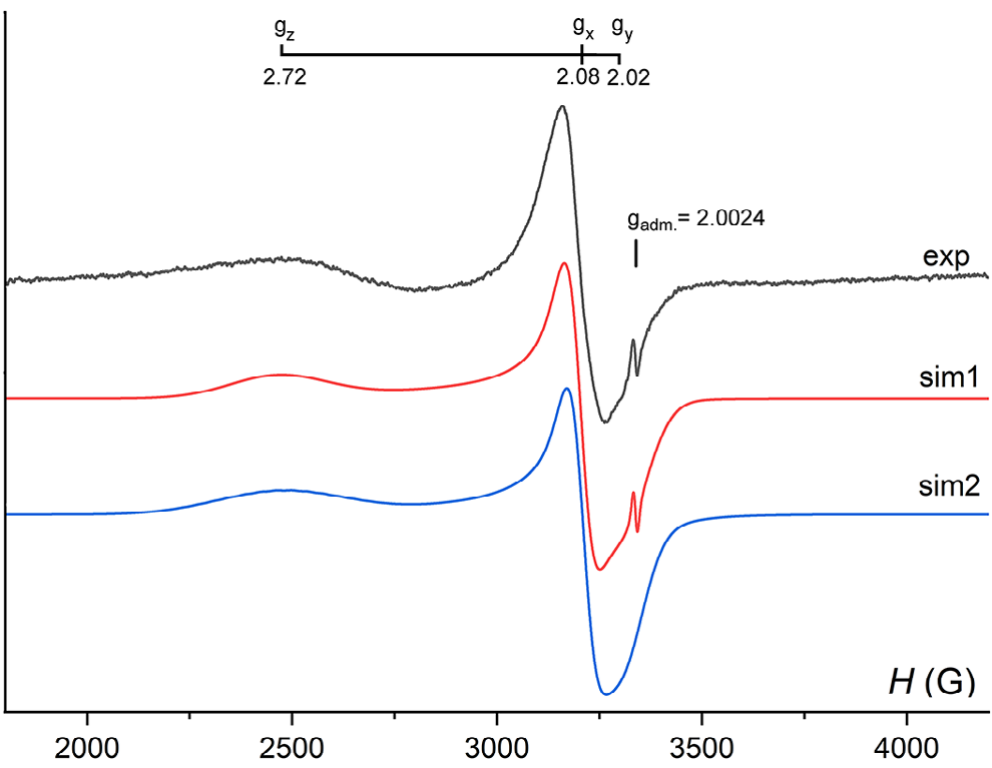
The experimental X‐band EPR spectrum of a thin amorphous film of **1** at 4 K (exp) and the best‐fit simulations using Easyspin 6.0.2 (sim1) and WINEPR Simfonia 1.25 (sim2). The simulation parameters are given in text and ESI.

Since electron counting considerations and electronic structure calculations both support that **1** is stable with an open shell, we performed X‐band EPR spectroscopic measurements for a solid sample of **1**. EPR measurements were earlier performed on the open‐shell gold clusters Au_25_ and Au_133_.^[^ ^31^ ^]^ At higher temperatures, due to known artifacts of the instrument and the quartz glass, only a small signal was observed for **1**. At a temperature of liquid helium (4 K), an anisotropic EPR signal typical for systems with *S* = 1/2 is observed (Figure 7 (exp)). The spectral pattern is simulated using the Easyspin 6.0.2 package (Figure 7 (sim1)); details are given in Section S8). Also, a minor (with a calculated content of less than 0.06%) paramagnetic impurity with *g* = 2.0024 was observed in the experimental spectrum and taken into account in the simulation (Figure 7 (sim1)) as a signal with *g*_adm._). In contrast to solid‐state EPR spectra of the clusters Au_25_(S‐CH_2_CH_2_Ph)_18_^[^ ^32^, ^33^, ^34^ ^]^ or [Au_25_(PPh_3_)_10_(SePh)_5_Cl_2_]^+[^ ^35^ ^]^ which have a pronounced rhombic symmetry (*g_x_* ≠ *g_y_* ≠ *g_z_*) with *g*_x_ = 2.5–2.6, *g*_y_ = 2.3–2.4, and *g*_z_ = 1.8–1.9 or *g*_x_ = 2.40, *g*_y_ = 2.26, and *g*_z_ = 1.78, the EPR spectrum of **1** has nearly axial symmetry of *g*‐tensor: the simulation has given the *g*‐tensor values *g*_z_ = 2.72, *g*_x_ = 2.08, and *g*_y_ = 2.02, and is generally close to the X‐band EPR spectrum of gold cluster Au_133_(SPh*^t^* Bu)_52_ with axial anisotropy (with *g*_‖_ = 2.47 and *g*_⊥_ = 1.7–1.8).^[^ ^31^ ^]^

Worthy of note, the pattern of *g*‐tensor (*g*_‖_ > *g*_⊥_) is reversed in the case of Au_147_(SPh)_30_(PPh_3_)_12_ (if we consider *g*_z_ = 2.72 as *g*_‖_ and *g*_⊥_ = 2.05—an average of *g*_x_ and *g*_y_) as well as Au_133_(SR)_52_ in comparison with clusters of lower nuclearity (e.g., Au_25_) where (*g*_‖_ < *g*_⊥_). Despite the absence of observable hyper‐fine splitting (HFS) on magnetic gold nuclei in anisotropic EPR spectra of gold clusters reported earlier, the authors have performed DFT simulations of hyperfine coupling constants (*A*_iso_) and included this parameter in EPR simulation. In the case of Au_25_(SR)_18_ , the value *A*_iso_ on 12 gold atoms forming the icosahedral Au_12_ shell falls in the range of 36–56 (av. 48) MHz (approx. 13–20 G) and 1.5 MHz only on the central gold atom.^[^ ^32^ ^]^ M. Hendrich and R. Jin have applied the close HFS constant *A*_iso_ = 50 MHz on 12 equivalent gold nuclei for Au_133_(SR)_52_.^[^ ^31^ ^]^ In order to compare such simulations, we have performed a simulation of the anisotropic X‐band EPR spectrum of **1**, taking into account such HFS on ^197^Au isotopes (*I* = 3/2, μ(µN) = +0.145746, 100% abund.) using WINEPR Simfonia (Figure 7 (sim2)). The initial values of the HFS parameters used during the fitting process were taken from earlier reported HFS values.^[^ ^31^, ^32^ ^]^ We have not included the presence of minor paramagnetic impurity with *g*_adm._ = 2.0024 in this simulation (details are given in Supporting Information). The best fit simulation has used the *g*‐tensor components *g*_x_ = 2.085, *g*_y_ = 2.021, and *g*_z_ = 2.716 and components of T‐tensor *A*_x_ = 2 G (∼6.5 MHz), *A*_y_ = 5 G (∼16 MHz), and *A*_z_ = 38 G (∼128 MHz), giving the calculated isotropic hyperfine coupling constant *A*_iso_ of 51 MHz on 12 Au nuclei.

The attempts to simulate the spectrum with the use of a bigger number of gold nuclei (e.g., 42 as in the second Au_42_ shell), leads to a serious deterioration of the fittings. Thus, in conclusion, like in the case of other gold clusters Au_25_ and Au_133_, the spin density of the unpaired electron in **1** is mainly distributed on the first icosahedral Au_12_ shell.

EPR and DFT calculations show that **1** is a geometrically closed cluster with an electronically open shell. Hence, the geometric shell closure overcomes the preference for closing the electronic shells. In contrast, **2** is a geometrically open but electronically closed cluster. Given that the two clusters can be obtained from the same reaction in almost the same amount, it can be assumed that the preference to form a geometrically and electronically closed cluster is almost the same in this system. This provides evidence that the determining factors for the formation of a compound change significantly in this size range between clusters and nanoparticles.^[^ ^36^, ^37^ ^]^ However, to the best of our knowledge, there are no documented examples of a reaction exhibiting this behavior in a similar way, which provides a novel insight into the transition between molecules and nanoparticles in this system.

## Conclusion

The reduction of (Ph_3_P)AuSPh with L‐selectride yields a cocrystallizate of the two novel metalloid clusters Au_147_(SPh)_30_(PPh_3_)_12_ **1** and Au_146_(SPh)_30_(PPh_3_)_12_ **2**.^[^ ^38^ ^]^ The gold atoms in **1** are arranged in three geometrically closed icosahedral shells, each of which fulfills the Mackay criterion and gives 147 gold atoms. This makes **1** the first characterized triple‐shell icosahedral metalloid cluster. The ligand shell is formed by phosphines and thiolates. It is noteworthy that the thiolates do not form staple motifs, which is highly unusual for a thiolate‐stabilized gold cluster. This could be a consequence of the additional phosphine ligands. The odd number of electrons indicates that the geometrically closed cluster **1** is an electronically open shell system, a conclusion confirmed by both DFT calculations and EPR experiments. The second cluster **2** has an unoccupied atomic position in the outer shell of the gold core, unlike **1** with which it cocrystallizes, resulting in 146 gold atoms. Consequently, the second cluster is geometrically open but electronically closed due to the absence of one electron. The formation of these two clusters, driven by different factors—geometric for **1** and electronic for **2—**demonstrates that they are approaching the bulk limit for nanoscale systems, where both effects become similar. Consequently, they provide direct insight into this transition and offer a new understanding of the formation of metal clusters and the transition from molecules to bulk for metals in general.

## Supporting Information

The authors have cited additional references within the Supporting Information.^[^ ^39^, ^40^, ^41^, ^42^, ^43^, ^44^, ^45^, ^46^, ^47^, ^48^, ^49^, ^50^, ^51^, ^52^, ^53^, ^54^, ^55^, ^56^, ^57^ ^]^ The xyz‐files of the Au_146_ and Au_147_ clusters are attached Supporting Information.

## Conflict of Interests

The authors declare no conflict of interest.

## Supporting information



Supporting Information

Supporting Information

## Data Availability

The data that support the findings of this study are available in the supplementary material of this article.
