# Supplementary material for: Au147(SPh)30(PPh3)12: A Geometrically Closed, but Electronically Open Triple‐Shell Icosahedral Gold Cluster and its Geometrically Open Counterpart
Source: Angew Chem Int Ed Engl. 2025 May 16;64(26):e202500586. doi: 10.1002/anie.202500586 (PMC12184293; doi:10.1002/anie.202500586)
Supplement: Supplementary file 1 — Supporting Information [file ANIE-64-e202500586-s001.pdf]

Supporting Online Information for

$Au_{147}(SPh)_{30}(PPh_3)_{12}$: A Geometrically Closed, but Electronically Open Triple-Shell Icosahedral Gold Cluster and its Geometrically Open Counterpart

Markus Strienz[a], Andrei Poddelskii[a], Bridget K. Moll[b], Claudio Schrenk[a], Phillip Thomas[c], Andre Z. Clayborne[b], Andreas Schnepf[a,*]

[a] Chemistry Department, University of Tübingen, Germany

[b] Department of Chemistry & Biochemistry, George Mason University, Fairfax, VA USA 22030

[c] National Energy Research Scientific Computing Center (NERSC), Lawrence Berkeley National Laboratory, USA

1 Synthesis 2

1.1 Synthesis of $Ph_3PAuSPh$: 2

1.2 Synthesis of $Au_{146}(PPh_3)_{12}(SPh)_{30}$ **2**/$Au_{147}(PPh_3)_{12}(SPh)_{30}$ **1** 2

1.3 Synthesis of $Au_{11}(PPh_3)_8(SPh)_3$ **3** 2

2 Structural properties 3

2.1 Calculation of the packing density in **1** 3

2.2 Structural difference between Au_{146} and Au_{147} 4

3 EDX 5

4 SEM Images 6

5 DLS 9

6 Crystallographic Section 10

7 UV/Vis 13

8 EPR 14

9 Mass spectrometry 15

10 Computational Methods 16

11 Bibliography 18

1 Synthesis

All reactions were carried out at room temperature under inert conditions with argon.

1.1 Synthesis of $Ph_3PAuSPh$:

3.21 g (10 mmol) (THT)AuCl and 2.62 g (10 mmol) PPh_3 are dissolved in 100 ml THF. The reaction is stirred for two hours. The solvent is filtered and the remaining solid is extracted with DCM. The THF and the DCM solution are dried under vacuum to give Ph_3PAuCl.

4.94 g (10 mmol) Ph_3PAuCl is dissolved in 100 ml ethanol. 1.02 ml (10 mmol) HSPh and 1.94 ml (14 mmol) NEt_3 are added. The suspension is refluxed for two hours. The solvent is filtered and the remaining solid is washed twice with THF. The THF is removed by vacuum. The product can be crystallized from THF at -30 °C.

Both reactions are quantitative with almost 100 % yield.

1.2 Synthesis of $Au_{146}(PPh_3)_{12}(SPh)_{30}$ **2**/$Au_{147}(PPh_3)_{12}(SPh)_{30}$ **1**

0.568 g (1 mmol) $Ph_3PAuSPh$ is dissolved in 20 ml THF. 0.7 ml of 1 M L-Selectride solution in THF is slowly added with a syringe at room temperature. The colorless solution turns black almost immediately and is stirred for 16 hours. The solvent is removed by vacuum and the crude black solid is washed with pentane and extracted with benzene or toluene. While toluene results in faster crystallization in a three days, the slower crystallization in benzene (a few weeks) results in larger crystals.

Yield: 4 mg, (2% of the gold atoms)

1.3 Synthesis of $Au_{11}(PPh_3)_8(SPh)_3$ **3**

0.568 g (1 mmol) $Ph_3PAuSPh$ is dissolved in 20 ml THF. 0.7 ml of a 1 M L-Selectride solution in THF is slowly added with a syringe at 0 °C. The colorless solution turns black almost immediately and is stirred for 16 hours. The solvent is removed by vacuum and the crude black solid is washed with pentane and extracted with toluene. While the crystals of Au_{146}/Au_{147} are black, the crystals of Au_{11} are red and therefore easy to distinguish.

Yield: 15 mg, (4% of the gold atoms)

2 Structural properties

2.1 Calculation of the packing density in **1**

*Table S1: Calculation of packing density in of each icosahedron in **1**.*

Icosahedron	1	2	3
Radius gold atom [Å] [a]	1.3775	1.3858	1.3926
Volume of one gold atom [Å^3]	10.9431715	11.1421771	11.3070043
Number of goldatoms	3.5158	27.032	90.547
Total volume of all gold atoms	38.4740025	301.19533	1023.81532
Edge length icosahedron [b]	2.898	5.874	8.756
Volume icosahedron [c]	53.1067702	442.238525	1464.77866
Density	0.72446512	0.68106986	0.69895565
Literature [5]	0.72585	0.6976	0.69237

[a] The radius of the gold atoms is determined by the distances of the gold atoms along the fivefold axis.

[b] The edge length of the icosahedron is based on the distances of the molecule structure in the crystal.

[c] $V_{icosahedron} = 2.182*l_{edge}^3$

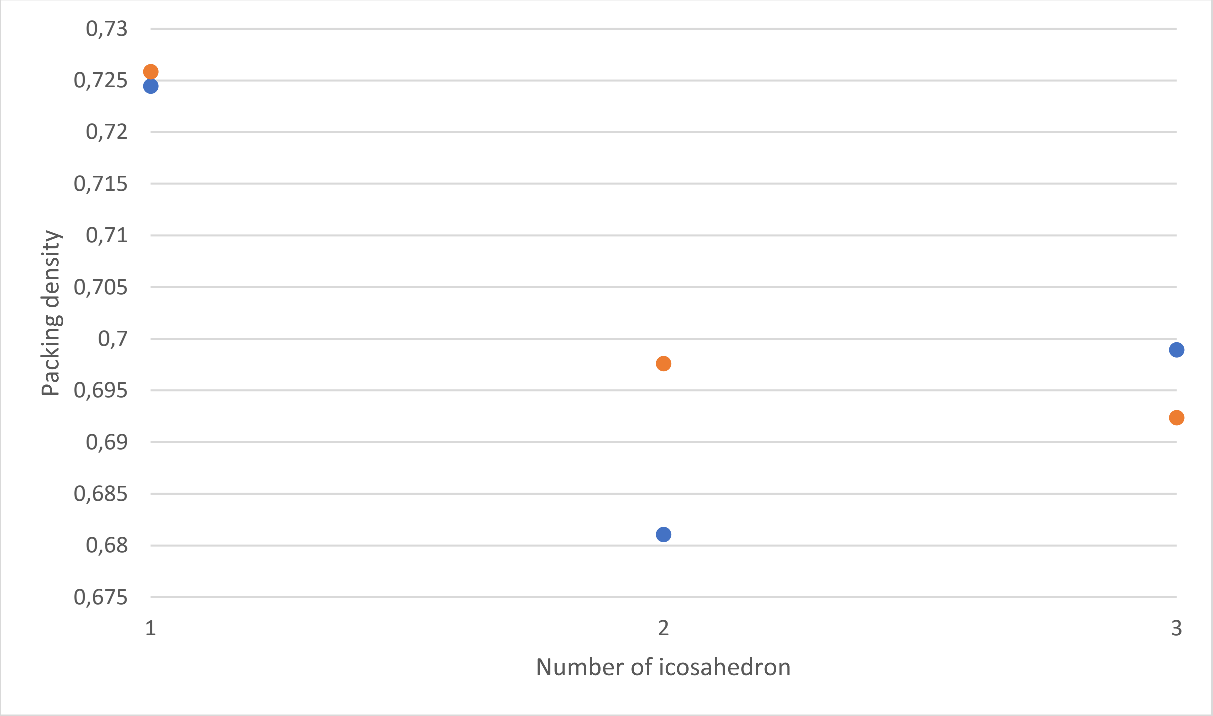

Figure S1: Comparison of the calculated values of the packing density of each icosahedron in 1(blue) compared to the packing density for the icosahedron with hard spheres (orange).

2.2 Structural difference between Au_{146} and Au_{147}

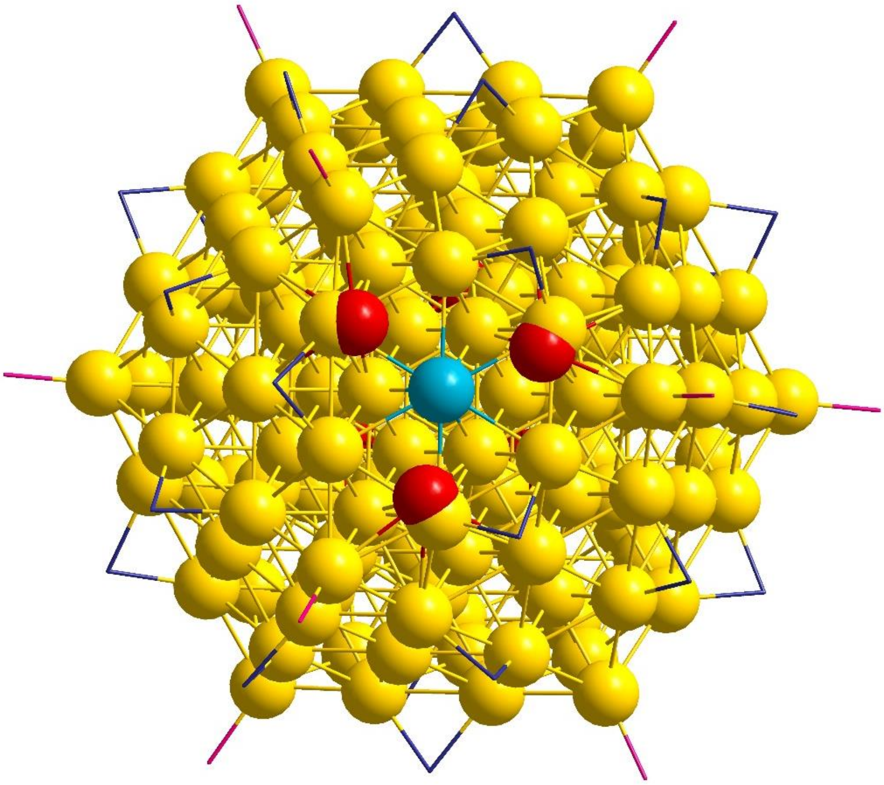

Figure S2: Image of 1/2. The unoccupied atom position is blue. The distorted gold atoms are colored in red.

3 EDX

*Table S2: Average atomic ratio for **1/2** compared to the calculated ratio.*

Element	Ratio (Au, S, P) measured [weight %]	Ratio (Au, S, P) calc. for **1** [weight %]	Ratio (Au, S, P) measured [Atom]	Ratio (Au, S, P) calc. for **1** [Atom]
Au	95.54 ± 0,78	95.6	146.43 ± 1.2	146.56
S	3.24 ± 0,66	3.17	30.48 ± 1.02	30
P	1.22 ± 0,35	1.23	11.9 ± 0.54	12

Nine points were measured by EDX. Each point is on a different crystal

Tabelle S3: Measured atomic ratio at point 5 compared to the calculated ratio.

Element	Abs. Ratio [weight %]	Ratio (Au, S, P) measured [weight %]	Ratio (Au, S, P) calc. for **1** [weight %]	Ratio (Au, S, P) measured [Atom]	Ratio (Au, S, P) calc. for **1** [Atom]
Au	87.44	96.57	95.6	148.05	146.56
S	2.92	3.2	3.18	30.201	30
P	1.03	1.13	1.23	11	12
C	27.91	-	-	-	-
O	2.03	-	-	-	-

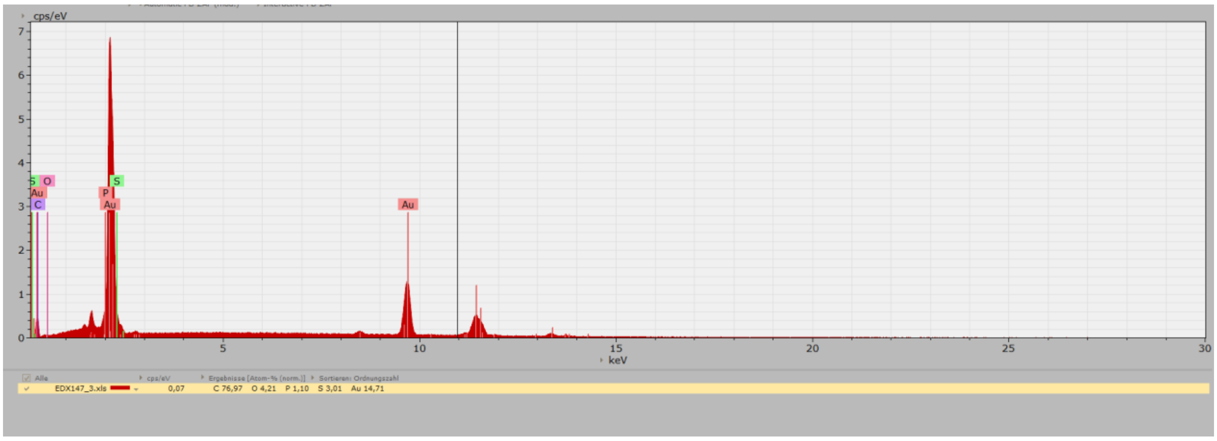

*Figure S3: EDX spectrum of **1/2**, measured at point 5.*

Table S1: EDX measurement parameters

Bruker Nano GmbH Berlin, Germany	
Date	29.05.2024
Primary energy	30 keV
Tilt angle	0 °
Take-off angle	30 °
Azimuth angle	0 °
Real time	120000 ms
Life time	113707 ms
Detector type	XFlash 6|60
Si layer	0.029 µm
Detector thickness	0.45 mm
Window type	slew AP3.3
Fano factor	0.112
Mn-FWHM:	127.1885 eV
Calibration, lin.	10 eV
Calibration, abs.	-955 eV
Channels	4096

4 SEM Images

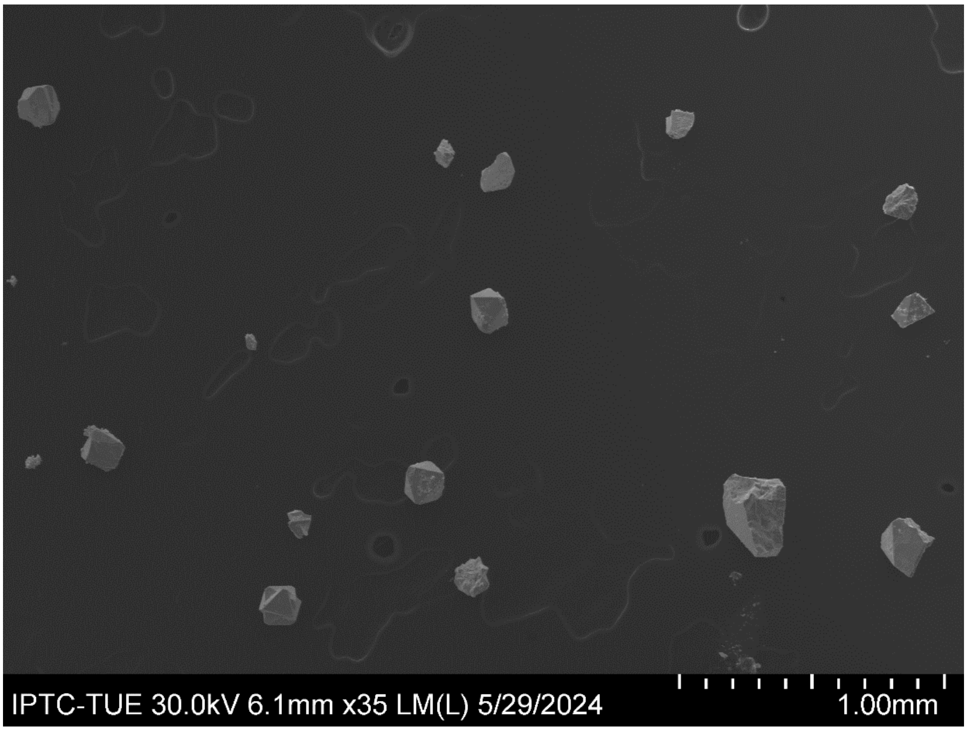

Figure S4: SEM image of crystals with 35x magnification.

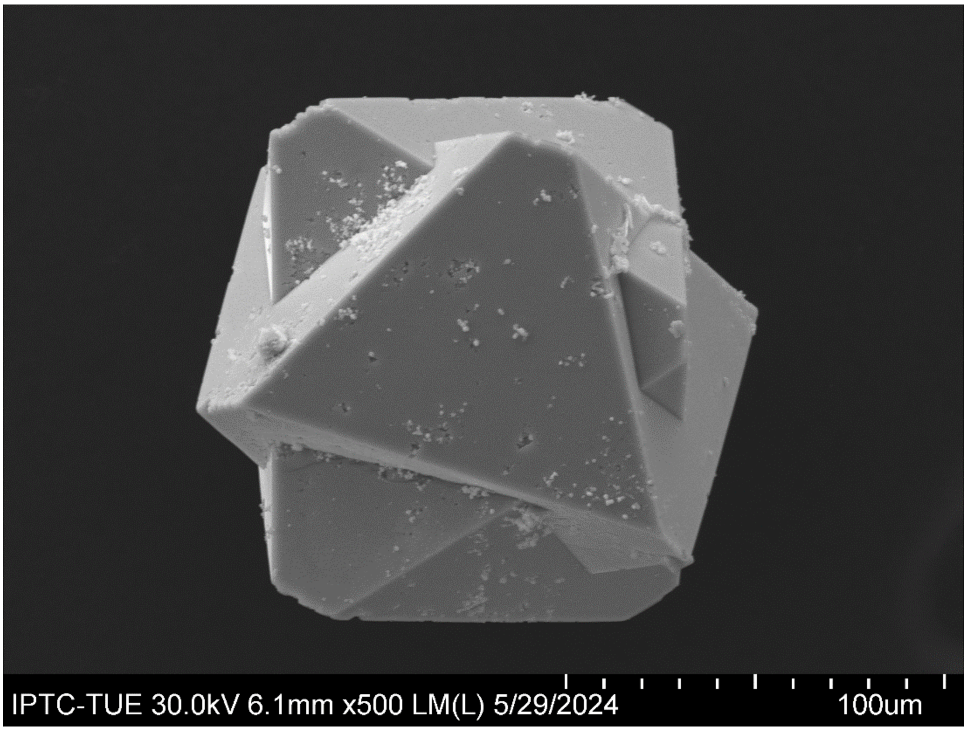

Figure S5: SEM image of a crystal with 500x magnification.

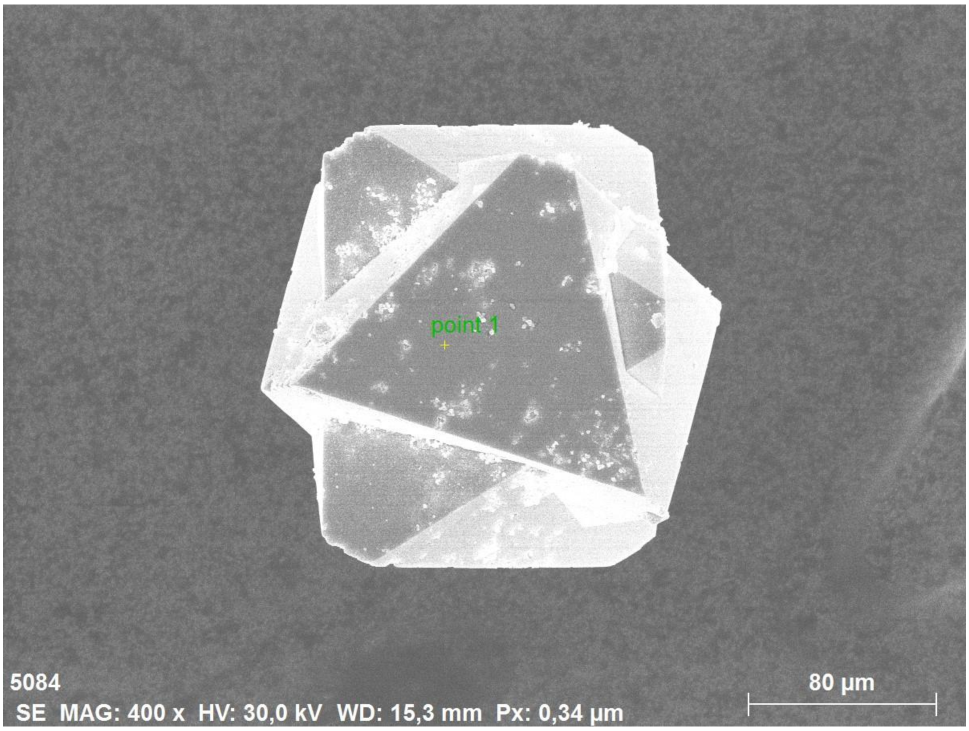

Figure S6: SEM image of a crystal with the point of EDX-measurement 1.

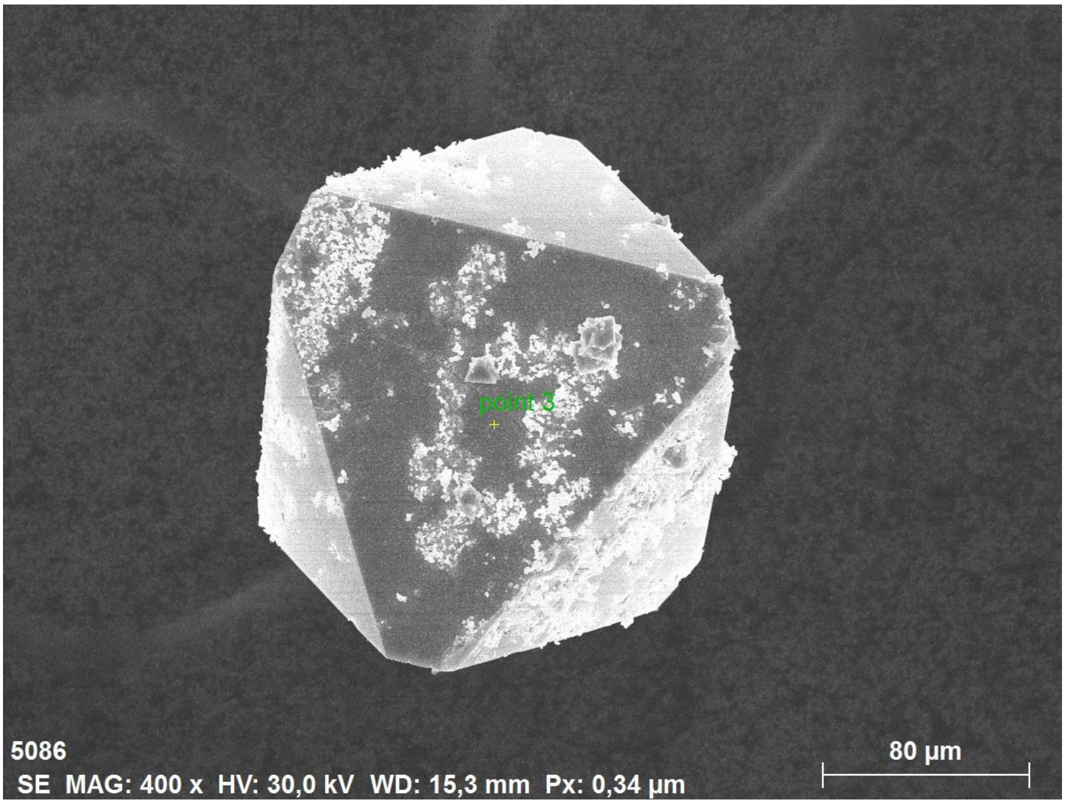

Figure S7: SEM image of a crystal with the point of EDX-measurement 3.

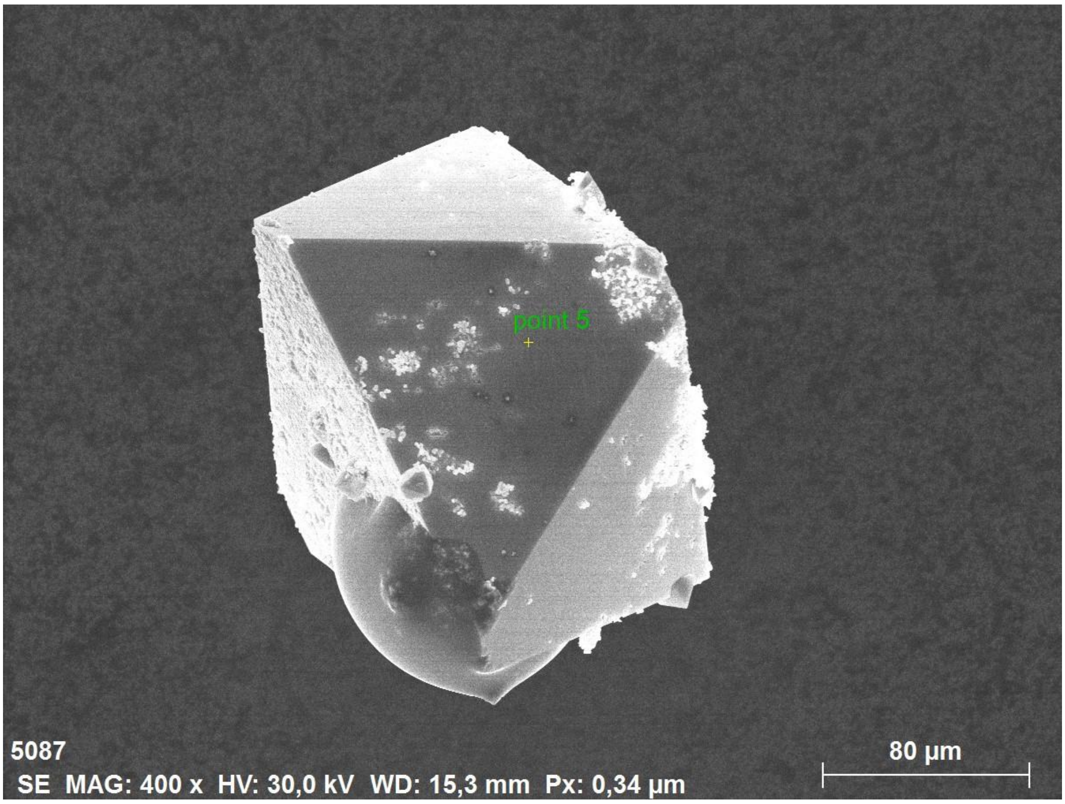

Figure S8: SEM image of a crystal with the point of EDX-measurement 5.

5 DLS

**Sample Name:**	Au147 1		
**SOP Name:**	mansettings.nano		
**File Name:**	Au147_DMI_3.dts	**Dispersant Name:**	DMI
**Record Number:**	7	**Dispersant RI:**	1.472
**Material RI:**	0.14	**Viscosity (cP):**	1.9500
**Material Absorbtion:**	0.000	**Measurement Date and Time:**	Montag, 27. Mai 2024 14:28:15

**Temperature (°C):**	25.0	**Duration Used (s):**	60
**Count Rate (kcps):**	358.4	**Measurement Position (mm):**	0.85
**Cell Description:**	Glass cuvette with round aperture	**Attenuator:**	10

			**Size (d.nm):**	**% Number:**	**St Dev (d.nm):**
Z-Average (d.nm):	64.69	**Peak 1:**	54.76	0.0	18.81
PdI:	0.487	**Peak 2:**	9.105	100.0	2.444
**Intercept:**	0.607	**Peak 3:**	0.000	0.0	0.000

Result quality : Good

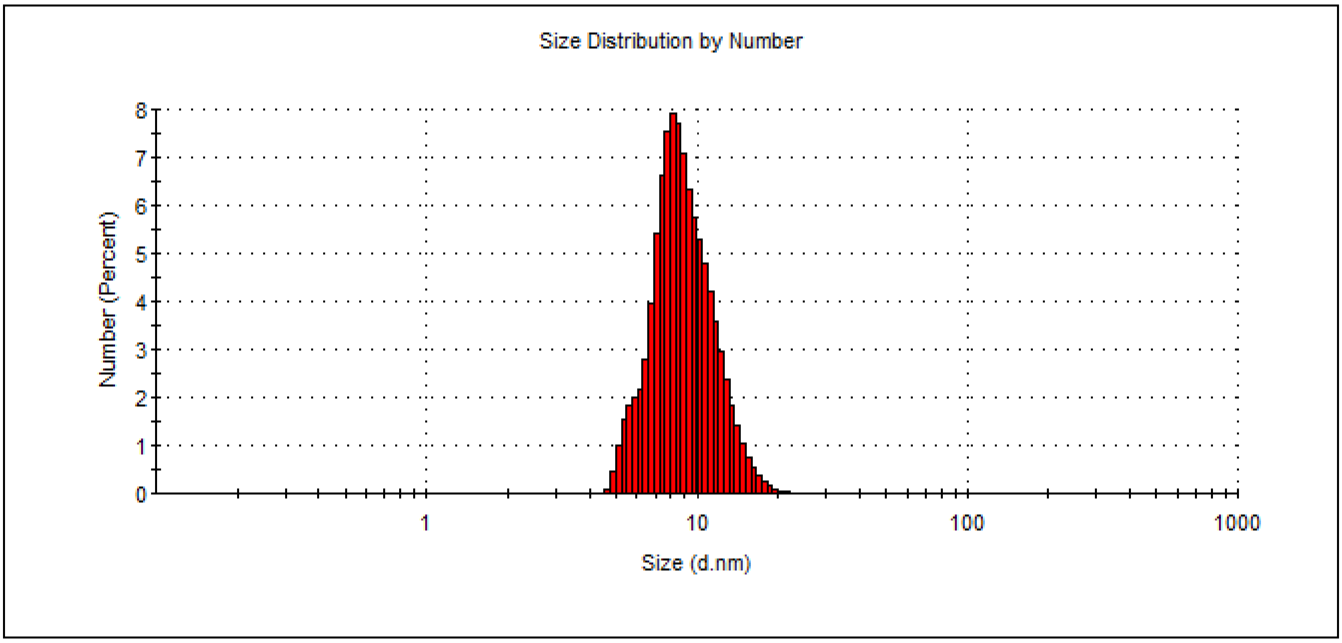

Figure S9: DLS settings and plotted size distribution by number.

6 Crystallographic Section

*Table S5: Crystallographic parameters of **1/2**.*

Empirical formula	$C_{396}H_{330}Au_{146.56}P_{12}S_{30}$
Formula weight	35288.78
Temperature/K	119.99(11)
Crystal system	trigonal
Space group	R-3
a/Å	25.95170(10)
b/Å	25.95170(10)
c/Å	65.5569(3)
α/°	90
β/°	90
γ/°	120
Volume/Å^3	38236.7(3)
Z	3
ρ_{calc}g/cm^3	4.598
μ/mm^{-1}	78.742
F(000)	44832.0
Crystal size/mm^3	0.073 × 0.06 × 0.043
Radiation	Cu Kα (λ = 1.54184)
2Θ range for data collection/°	4.768 to 130.166
Index ranges	-30 ≤ h ≤ 30, -30 ≤ k ≤ 30, -77 ≤ l ≤ 77
Reflections collected	227947
Independent reflections	14513 [R_{int} = 0.0658, R_{sigma} = 0.0187]
Data/restraints/parameters	14513/450/944
Goodness-of-fit on F^2	1.062
Final R indexes [I>=2σ (I)]	R_1 = 0.0590, wR_2 = 0.1743
Final R indexes [all data]	R_1 = 0.0681, wR_2 = 0.1872
Largest diff. peak/hole / e Å^{-3}	3.73/-2.95
CCDC number	2409434

Crystals of the Au_{146}/Au_{147} clusters were mounted on the diffractometer at 120 K. The data were collected on a Rigaku XtaLAB Synergy-S X-ray diffractometer for single crystal X-ray diffraction, equipped with a PhotonJet-S source for for monochromated CuKα radiation (λ = 1.54184 Å) and equipped with an Oxford Cryosystems cryostat. A numerical absorption correction based on gaussian integration over a multifaceted crystal model was applied as implemented in the program package CrysAlisPro. The structure was solved by direct methods and refined against F^2 for all observed reflections. Programs used: SHELXT and SHELXL [6, 7] within the Olex2 program package.[8]

The molecular structure could be best refined as a superposition of two Au_{146} clusters into each other via the pseudo symmetry element inversion. Since the arrangement of the gold atoms in the cluster core has only C_{5v} point group symmetry with one missing face centering atom, the location of Au1 at (1/3, 2/3, 2/3) in $R\overline{3}$ space group requires an inversion center. Therefore, the cluster is refined as an icosahedron with two perpendicular half-occupied triangular face centers, which is the of the pseudo D_{3d} point group symmetry. This superposition is underlined by a clearly identified split position of the next Au-S(Ph)-Au subunits, which could be properly refined as a 1:1 split model. The SQUEEZE [9] program routine is used to identify non-refineable solvent molecules. In this case, three voids with a volume of 1733 Å^3 each and 967 and 972 electrons could be identified. This fits to 23 heavily disordered benzene molecules per void, resulting in 69 benzene molecules per unit cell. To avoid problems with the SPh and PPh_3 groups, some phenyl rings were restrained with AFIX implemented in the routine FragmentDB.[10, 11]

The H atom positions in all compounds were refined using a riding model.

The supplementary crystallographic data can be obtained online free of charge at www.ccdc.cam.ac.uk/conts/retrieving.html or from Cambridge Crystallographic Data Centre, 12 Union Road, Cambridge CB21EZ; Fax: (+44)1223-336-033; or deposit@ccdc.cam.ac.uk.

Table S6: Crystallographic parameters of ***3****.*

Empirical formula	$C_{151}H_{128}Au_{11}P_7S_3$
Formula weight	4422.13
Temperature/K	149.99(10)
Crystal system	orthorhombic
Space group	$P2_12_12_1$
a/Å	16.61080(10)
b/Å	24.9931(2)
c/Å	31.6461(2)
α/°	90
β/°	90
γ/°	90
Volume/Å^3	13138.05(16)
Z	4
ρ_{calc}g/cm^3	2.236
μ/mm^{-1}	12.415
F(000)	8224.0
Crystal size/mm^3	0.12 × 0.108 × 0.054
Radiation	Mo Kα (λ = 0.71073)
2Θ range for data collection/°	3.91 to 56.564
Index ranges	-22 ≤ h ≤ 22, -33 ≤ k ≤ 33, -42 ≤ l ≤ 42
Reflections collected	363942
Independent reflections	32603 [R_{int} = 0.0519, R_{sigma} = 0.0252]
Data/restraints/parameters	32603/36/1550
Goodness-of-fit on F^2	1.062
Final R indexes [I>=2σ (I)]	R_1 = 0.0219, wR_2 = 0.0358
Final R indexes [all data]	R_1 = 0.0252, wR_2 = 0.0364
Largest diff. peak/hole / e Å^{-3}	1.02/-0.68
CCDC number	2409433

Crystals of the Au_{11} clusters were mounted on the diffractometer at 150 K. The data were collected on a Rigaku XtaLAB Synergy-S X-ray diffractometer for single crystal X-ray diffraction, equipped with a PhotonJet-S source for for monochromated MoKα radiation (λ = 0.71073 Å) and equipped with an Oxford Cryosystems cryostat. A numerical absorption correction based on gaussian integration over a multifaceted crystal model was applied as implemented in the program package CrysAlisPro. The structure was solved by direct methods and refined against F^2 for all observed reflections. Programs used: SHELXT and SHELXL [6, 7] within the Olex2 program package.[8]

7 UV/Vis

The UV/Vis data were collected on a PG INSTRUMENTS LIMITED T60 spectrometer.

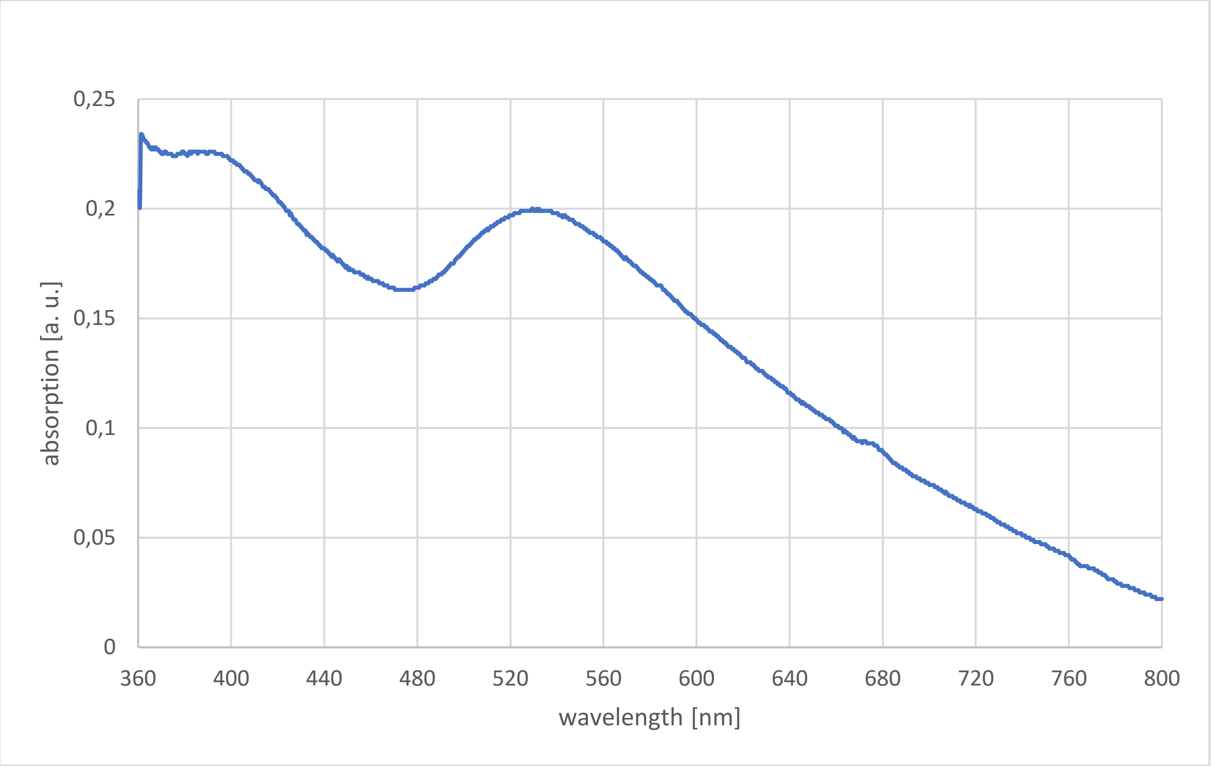

Figure S10: Absorption spectrum of ***1/2*** *in DMI.*

8 EPR

For the EPR measurements, the sample was dissolved in THF and filled into quartz glass EPR tubes. The solvent was then removed under vacuum, and a thin film of Au_{146}/Au_{147} was obtained. The X-band EPR spectrum of $Au_{147}(SPh)_{30}(PPh_3)_{12}$ was measured on a Bruker ELEXSYS spectrometer equipped with a helium continuous-flow cryostat (Oxford CF935) and a variable temperature controller unit (Oxford ITC-4) at 4 K using the thin film sample. The experimental conditions: Frequency 9.355 GHz, Modulation frequency 100 kHz, Modulation amplitude 3 G, Field scan rate 1.22 G s^{-1}.

Simulation sim1 of EPR spectrum was performed with the MATLAB tool EasySpin 6.0.2.[12] The following simulation parameters give the best fit: g_x = 2.084, g_y = 2.020, g_z = 2.72; HStrain of 20, 340, and 1070 MHz, respectively, for unresolved HFS on each spectral component; an inhomogeneous broadening due to g distribution (gstrain ±0.0066, ±0.0066, and ±0.015, respectively); lw_1 = [6 1] mT; admixture of minor impurity: g = 2.0024, lw = 0.93 mT, weight = 0.0006.

Simulation sim2 of EPR spectrum was performed with WINEPR Simfonia 1.25.[13] The following best fitting parameters were achieved: g_x = 2.085, g_y = 2.021, g_z = 2.716; A_x = 2 G, A_y = 5 G, A_z = 38 G (A_{iso} = 15 G, or approx. 51 MHz); linewidths are 55, 90, and 40 G; Lorentzian/Gaussian = 0.9.

Also, we have to note, J.A. Gascón and F. Maran have shown that the EPR spectrum of Au_{25} cluster may be reproduced without a necessary to use HFS on all 12 (13 with a central Au nucleus) ^{197}Au nuclei: smaller numbers of equivalent gold atoms affect weakly the simulation pattern due to the broaden anisotropic hyperfine structure.[14] In our case, the simulations sim1 and sim2 are very similar and provide a qualitative fit to the experimental EPR spectrum, nevertheless the high field component with g_z = 2.72 in sim2 tends to fit better the experimental spectrum pointing out the presence of proposed HFS on 12 gold nuclei.

9 Mass spectrometry

For the mass spectrometry measurements, 10 mg of crystals of Au_{146}/Au_{147} were dissolved in 10 ml THF. The ESI-TOF measurements were performed on an Acquity UPLC / Synapt G2 HDMS from waters. Additional samples were measured after the addition of a solution of caesium acetate in THF as well as a solution of caesium acetate in acetonitrile.

Instrument Configuration:

Lteff	1800.0
Veff	7221.23
Resolution	10000
Min Points in Peak	2
Acquisition Device	WatersADC
ADC Trigger Threshold (V)	1.60
ADC Input Offset (V)	-1.71
Average Single Ion Intensity	20
ADC Amplitude Threshold	3
ADC Centroid Threshold	-1
ADC Ion Area Threshold	2
ADC Ion Area Offset	10
ADC Pushes Per IMS Increment	1
EDC Delay Coefficient	1.5700
EDC Delay Offset	1.8100
Tof Emulation Transfer Pulse Height (V)	0.1

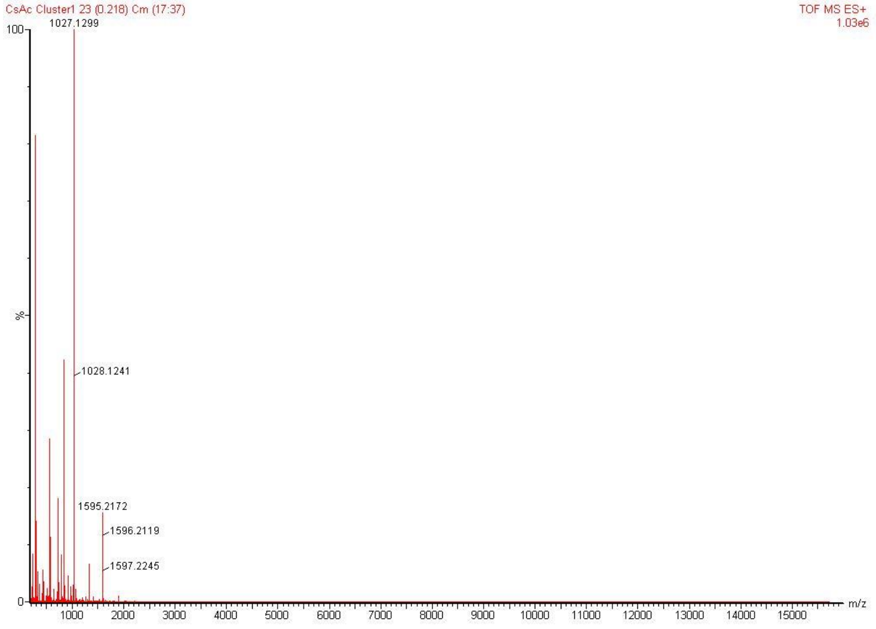

Figure S11: Mass spectrum of a solution of Au_{146}/Au_{147} in THF with caesium acetate as an additive.

10 Computational Methods

Density functional theory (DFT) calculations were carried out using Quantum ESPRESSO (QE) and additional calculations were carried out using tight binding DFT in the AMS DFTB 2024.1 engine to obtain electronic structure information.[15] QE calculations were carried out using the generalized-gradient approximation (GGA) exchange-correlation with the Perdew-Burke-Ernzerhof (PBE) functional for Au_{146} and Au_{147}, a common functional for gold clusters.[16, 17] The crystal structure was used, however the phosphine was modified by only using the hydrogen instead of the benzene derivative of the crystal structure. The wavefunction kinetic energy cutoff was set to 40.0 Ry and charge density kinetic energy cutoff was 300.0 Ry, both values being the lowest within the normal range for medium sized gold nanoparticles. We used ultrasoft pseudopotentials to describe the electron-ion interactions. The total energy convergence threshold was set to 10^{-5} eV/Bohr and the force convergence threshold was set to 10^{-4} eV/Bohr. The Bravais-lattice index was set to zero for a free geometry with a 32.0 Bohr box with an additional 10 Bohr of vacuum space in all directions. The nosym_evc field was set to true to allow the k points symmetry to be equal to the Bravais lattice settings. It was necessary to increase the maximum number of iterations per scf step to 300, to ensure convergence was reached, the maximum stress on an atom, was 10^{-8} eV/Å, with Broyden mixing mode. For the DFTB calculations, we used the self-consistent charge formalism with DFTB.org auorgap-1-1 for the atoms of both **1** and **2**.[18-21] The charge convergence was set to 1 x 10^{-9}. For calculations in both QE and DFTB, the simulations were carried out without geometry optimization to obtain gaps and electronic structure.

The gap for Au_{146} was 0.091 eV using QE and 0.082 using DFTB. These gaps are within 0.009 eV of one another. The gap of **1** and **2** was determined by taking the difference between the lowest unoccupied and highest occupied molecular orbital. Table S1 displays HOMO-LUMO gap values for various size AuNPs. The HOMO-LUMO gap for Au146 of 0.091 eV is smaller than other AuNPs of greater than 100 Au atoms, specifically Au_{103}, Au_{108}, Au_{133}, and Au_{144}. The small gap and minimal change in gap when

adding or removing the central atom, may be an indicator that the Au_{146} / Au_{147} clusters are approaching the bulk limit for nanoscale systems with both thiol and phosphine ligands.

Table S7: DFT determined gaps for various AuNPs.[3, 21-25] Au_2 and Au_{11} are for clusters without ligands.

AuNP	HOMO-LUMO Gap (eV)	Reference
Au2	2.127	[22]
Au11	1.099	[22]
Au103	0.450	[24]
Au108	0.680	[21]
Au133	0.110	[3]
Au144	1.670	[25]
Au146	0.082 (0.091)	This Work*
Au147	0.005	This Work[#]

*The value in parenthesis was determined using QE

[#]Only the DFTB value is reported.

11 Bibliography

[1] C. E. Briant, B. R. C. Theobald, J. W. White, L. K. Bell, D. M. P. Mingos, A. J. Welch, *J. Chem. Soc., Chem. Commun.* **1981**, (5), 201.
[2] N. Yan, N. Xia, L. Liao, M. Zhu, F. Jin, R. Jin, Z. Wu, *Sci. Adv.* **2018**, *4* (10), eaat7259.
[3] A. Dass, S. Theivendran, P. R. Nimmala, C. Kumara, V. R. Jupally, A. Fortunelli, L. Sementa, G. Barcaro, X. Zuo, B. C. Noll, *J. Am. Chem. Soc*. **2015**, *137* (14), 4610-4613.
[4] J. Yan, S. Malola, C. Hu, J. Peng, B. Dittrich, B. K. Teo, H. Häkkinen, L. Zheng, N. Zheng, N. *Nat. Commun.* **2018**, *9* (1), 3357.
[5] A. Mackay, *Acta Crystallogr.* **1962**, *15* (9), 916-918.
[6] G. Sheldrick, *Acta Cryst. Sec. A* **2008**, *64*, 112–122.
[7] G. M. Sheldrick, *Acta Cryst.* **2015**, *71*, C3–C8.
[8] O. V. Dolomanov, L. J. Bourhis, R. J. Gildea, J. A. K. Howard, H. Puschmann, *J. Appl. Crystallogr.* **2009**, *42*, 339–341.
[9] A. L. Spek, *Acta Crystallogr. D Biol. Crystallogr.* **2009**, *65*, 148–155.
[10] D. Kratzert, J. J. Holstein, I. Krossing, *J. Appl. Crystallogr.* **2015**, *48*, 933–938.
[11] D. Kratzert, I. Krossing, *J. Appl. Crystallogr.* **2018**, *51*, 928–934.
[12] S. Stoll, A. Schweiger, *J. Magn. Reson.* **2006**, *178*, 42–55.
[13] Bruker Analytische Messtechnik GmbH, WINEPR Simfonia 1.25 **1996**.
[14] S. Antonello, N. V. Perera, M. Ruzzi, J. A. Gascón, F. Maran, *J. Am. Chem. Soc.* **2013**, *135*, 15585–15594.
[15] R. Rüger, A. Y., P. Philipsen, S. Borini, P. Melix, A. F. Oliveira, M. Franchini, T. van Vuren, T. Soini, M. de Reus, M. G. Asl, T. Q. Teodoro, D. McCormack, S. Patchkovskii, T. Heine, AMS DFTB 2024.1, SCM. Theoretical Chemistry, Vrije Universiteit, Amsterdam, The Netherlands **2024**.
[16] M. J. Cowan, G. Mpourmpakis, *Nanoscale Adv.* **2019**, *1*, 184–188.
[17] Y. G. Srinivasulu, Q. Yao, N. Goswami, J. Xie, *Mater. Horiz.* **2020**, *7*, 2596–2618.
[18] M. Elstner, D. Porezag, G. Jungnickel, J. Elsner, M. Haugk, T. Frauenheim, S. Suhai, G. Seifert, *Phys. Rev. B* **1998**, *58*, 7260–7268.
[19] T. A. Niehaus, S. Suhai, F. Della Sala, P. Lugli, M. Elstner, G. Seifert, T. Frauenheim, *Phys. Rev. B* **2001**, *63*, 085108.
[20] A. Fihey, C. Hettich, J. Touzeau, F. Maurel, A. Perrier, C. Köhler, B. Aradi, T. Frauenheim, *J. Comp. Chem.* **2015**, *36*, 2075–2087.
[21] V. Q. Vuong, J. M. L. Madridejos, B. Aradi, B. G. Sumpter, G. F. Metha, S. Irle, *Chem. Sci.* **2020**, *11*, 13113–13128.
[22] J. Wang, G. Wang, J. Zhao, *Phys. Rev. B* **2002**, *66*, 035418.
[23] T. Iqbal, A. Azam, A. Majid, M. Zafar, M. Shafiq, S. Ullah, M. Hussien, *Opt. Quantum Electron.* **2022**, *54*, 74.
[24] T. Higaki, C. Liu, M. Zhou, T.-Y. Luo, N. L. Rosi, R. Jin, *J. Am. Chem. Soc.* **2017**, *139*, 9994–10001.
[25] Z. Lei, J.-J. Li, X.-K. Wan, W.-H. Zhang, Q.-M. Wang, *Angew. Chem. Int. Ed.* **2018**, *57* (28), 8639-8643.
